# Prostate cancer incidentally discovered at the time of radical cystoprostatectomy does not decrease overall survival: Results from a large Chinese medical center

**DOI:** 10.1590/S1677-5538.IBJU.2017.0430

**Published:** 2018

**Authors:** Shiying Tang, Han Hao, Dong Fang, Wei Zheng, Peng Ge, Xiaohong Su, Qun He, Xinyu Yang, Qi Shen, Xuesong Li, Wei Yu, Jian Lin, Liqun Zhou

**Affiliations:** 1 Department of Urology, Peking University First Hospital, Institute of Urology, Peking University, National Urological Cancer Center, Beijing, China

**Keywords:** Urinary Bladder Neoplasms, Prostatic Neoplasms, Carcinoma

## Abstract

**Purpose:**

To investigate the incidence and pathologic characteristics of prostate cancer (PCa) incidentally discovered at the time of radical cystectomy and its impact on overall survival.

**Materials and Methods:**

A single center retrospective study of 762 male patients who underwent radical cystoprostatectomy from Jan 1994 to Dec 2012.

**Results:**

Of all included patients, 132 (17.3%) were found to have PCa. Patients with incidental PCa had a significantly higher mean age (69.2 vs. 62.2 years, P=0.015). Among the 132 patients with PCa, prostate specific antigen (PSA) analysis was available in 76 patients (57.6%), with a median value of 1.06ng/mL, and 61 (80.3%) patients had a PSA value below 4ng/mL. Four hundred and thirty-six patients (57.1%) were successfully followed, with a median duration of 46.5 months. The overall 5-year survival rate was 62.1%, and the 5-year cancer–specific survival rate was 72%. PCa recurrence was defined by two consecutive PSA values of >0.2 ng/mL and rising, and no PCa recurrence occurred. According to a univariate analyses, incidental PCa was not associated with cancer-specific survival (P=0.192) or overall survival (P=0.493). According to univariate analyses, the overall survival of patients with PCa was not associated with prostate cancer staging, PSA value, or Gleason score (All P values>0.05).

**Conclusions:**

Prostate cancer incidentally discovered at the time of radical cystectomy does not decrease overall survival. Patients with incidental PCa were older than those without. The PSA value before operation is not helpful for predicting incidental prostate cancers.

## INTRODUCTION

Prostate cancer (PCa) in China does not occur as often as in the Western world. However, in recent years, the incidence of this disease has continuously grown. The prevalence of latent PCa has been reported to be much higher in autopsy studies (1). Carcinoma of the bladder is the most common malignancy of the urinary system in China. Many patients have been diagnosed with muscle-invasive disease or high-risk non-muscle invasive bladder cancer. Radical cystoprostatectomy with bilateral pelvic lymph node dissection is the standard treatment for these patients (2). To preserve sexual function in selected cases, alternative techniques have been reported and some authors have described cystectomy with partial prostatectomy (3, 4). Concerns have been raised about the possible association with incidental prostate cancer, which may lead to potential risks.

In a previous publication from our center, Yang et al. had reported that the rate of incidental PCa was 28% (5). It is notable that in China, data regarding the impact of incidentally discovered prostate cancer at the time of radical cystoprostatectomy on overall survival is still lacking. Thus, we updated our database and performed this study. We aim to investigate not only the incidence and pathological characteristics of incidentally discovered prostate cancer in radical cystoprostatectomy specimens but also its impact on patients’ survival.

## MATERIALS AND METHODS

### Patient Selection

From Jan 1994 to Dec 2012, a total of 763 male patients underwent radical cystoprostatectomy at Peking University First Hospital. Selection criteria included a) no previous history of prostate cancer; b) no previous history of chemotherapy or radiotherapy; and c) no evidence of prostate cancer in imaging; d) age ≥40 years old. A total of 762 patients were included in this study. It is notable that we excluded one patient due to a pre-operative PSA value of 136ng/mL, and later results showed that he had pT4 prostate cancer with a Gleason score of 5+5. Clinical data were retrospectively collected. The preoperative evaluation included digital rectal examination, contrast-enhanced computed tomography (CT), and prostate specific antigen (PSA). This study was approved by the institutional review board.

### Patients treatment and follow-up

All patients were treated with *en bloc* open or laparoscopic cystoprostatectomy with or without bilateral pelvic lymph node dissection. Lymph node dissection included all the lymph nodes in the boundaries of the: common iliac bifurcation (cephalad); genitofemoral nerve (laterally); circumflex iliac vein and lymph node of Cloquet (caudal); hypogastric vessels (posteriorly), including the obturator fossa. Surgical margin of the urethra was not routinely examined through frozen biopsy.

All patients were followed up every three months for the first two years after operation and every six months in the following periods. During follow-up, chest X-ray was used to detect metastasis in the chest cavity, CT scan was used to detect abdominal metastasis and local recurrence. For patients with prostate cancer, PSA was routinely monitored at three months intervals. PCa recurrence was defined by two consecutive PSA values of >0.2ng/mL and rising in our study.

### Pathologic evaluation

All pathology specimens were reviewed by three experienced pathologists at our institution. The prostates of all patients were embedded and sectioned at 3-mm intervals. Prostate cancers found in the specimens were assessed for stage, surgical margin status and Gleason score. 2005 International Society of Urological Pathology (ISUP) Modified Gleason System was used for the grading of PCa. Pathological tumor staging for bladder cancer and prostate cancer were based on the 2010 TNM classification of the American Joint Committee on Cancer.

### Statistical analysis

Data were retrospectively collected at our center. Continuous variables were compared between groups using a t test. Descriptive statistics were performed using Pearson's chi--squared test. Kaplan-Meier plots were used to estimate overall survival and cancer-specific survival, and differences were assessed with the log-rank statistic. Multivariate logistic regression was used to calculate the predictive factors. Multivariate Cox regression analysis was used to evaluate the prognostic factors for survival. Prostate cancer recurrence includes PSA recurrence (defined as a PSA value over 0.2ng/mL), local recurrence and distant metastases. SPSS 17.0 was used for the data analysis. The level of statistical significance was set at *P*<0.05. All P-values are two sided.

## RESULTS

Of all 762 patients included in this study, 697 had urothelial carcinoma, while the other 65 patients had other pathologic subtypes (13 adenocarcinomas, 11 squamous cell carcinomas, 9 sarcomas, and 32 other types). The mean age of the patients was 63.5 years (range, 41-91 years). Among the patients, 2 had Ta disease, 210 had T1 disease, 218 had T2 disease, 168 had T3 disease, 128 had T4 disease, and 6 had CIS only. Lymph node dissection was performed in 503 patients, while 109 (21.67%) patients showed lymph node involvement.

Of all patients included, 132 (17.3%) were found to have incidental prostate cancer. The mean age of the patients with prostate cancer was 69.2 years (range 44-87 years). There was a significant difference between the age of the patients with and without prostate cancer (69.2 vs. 62.2 years, *P*=0.015). Table-1 lists bladder tumor stage and lymph node status in each group, all of which showed no significant difference. According to a univariate analysis, only elder age was related to the presence of incidental prostate cancer (HR=1.068, 95% confidence interval [CI] 1.046-1.091, *P*<0.001). Positive margin of prostate cancer was noted in one patient; this patient had a Gleason score of 3+3, a PSA value of 4.25ng/mL, and the pathologic stage was pT2a.

**Table 1:** Comparison of clinical and pathologic characteristics between patients with or without incidental prostate cancer.

		Patients without prostate cancer	Patients with prostate cancer	Total	Chi-square	P value
Age, year, mean		69.2	62.2			0.015
**Bladder tumor stage**						
	pT1	186	35	210		
	pT2	178	50	218		
	pT3	148	30	168		
	pT4	111	17	128		
	pTa	2	0	2		
	CIS	6	0	6		
	**Total**	**630**	**132**	**762**	**7.659**	**0.176**
**Lymph node status**						
	Negative	310	84	394		
	Positive	93	16	109		
	**Total**	**403**	**100**	**503**	**4.388**	**0.223**

PSA analysis was available for 76 of the 132 patients with prostate cancer (57.6%), with a median value of 1.06ng/mL (range 0.02-19.37ng/mL), and 61 (80.3%) patients had a PSA value below 4 ng/mL. Clinical and pathologic characteristics for patients with prostate cancer are listed in Table-2. Fifteen patients were found to have a PSA value over 4ng/mL; detailed information on these patients is listed in Table-3.

**Table 2:** Clinical and pathologic characteristics for patients with prostate cancer.

Variables		Number of patients (%)	Number of patients with pre-operative PSA value	Mean PSA value (ng/mL)	P value
**Age**					
	40-49	4 (3.03)	2	0.58	
	50-59	18 (13.64)	8	2.64	
	60-69	39 (29.55)	21	2.57	
	70-79	59 (44.70)	37	6.26	
	≥80	12 (9.09)	9	3.81	0.906
**Tumor stage**					
	pT2a	94 (71.21)	51	2.05	
	pT2b	1 (0.76)	0		
	pT2c	32 (24.24)	20	3.50	
	pT3a	4 (3.03)	4	6.40	
	pT3b	0 (0)	0		
	pT4	1 (0.76)	1	5.99	0.556
**Gleason score**					
	3+2	40 (30.30)	14	1.75	
	3+3	65 (49.24)	24	1.42	
	3+4	22 (16.67)	17	3.46	
	4+3	3 (2.27)	2	10.01	
	4+4	1 (0.76)	1	5.09	
	5+5	1 (0.76)	1	5.99	<0.001
**Gleason score in each risk group**					
	<7	105 (79.5)	55	2.11	
	=7	25 (18.9)	19	4.15	
	>7	2 (1.5)	2	5.54	0.053

**Table 3:** Detailed information on patients with a PSA value over 4ng/mL.

	PSA value (ng/mL)	Gleason score	Pathologic stage	Margin status	Follow-up result*
1	4.25	3+3	pT2a	Positive	Lost
2	4.45	3+4	pT2c	Negative	Dead
3	5.09	4+4	pT3a	Negative	Survive
4	5.21	3+3	pT2a	Negative	Lost
5	5.60	3+4	pT2c	Negative	Dead
6	5.82	3+3	pT2a	Negative	Dead
7	5.99	5+5	pT4	Negative	Dead
8	6.40	3+3	pT2a	Negative	Dead
9	7.79	3+4	pT2c	Negative	Survive
10	8.81	3+2	pT2a	Negative	Survive
11	9.24	3+4	pT2a	Negative	Dead
12	11.06	3+3	pT2a	Negative	Survive
13	12.34	3+3	pT2c	Negative	Dead
14	15.30	3+4	pT2c	Negative	Survive
15	19.37	4+3	pT3a	Negative	Survive

* For the 15 patients who had a PSA value over 4 ng/mL, 13 patients were successfully followed, 7 died of bladder cancer, and the other 6 survived and were free of either bladder cancer or prostate cancer.

**Figure 1:**
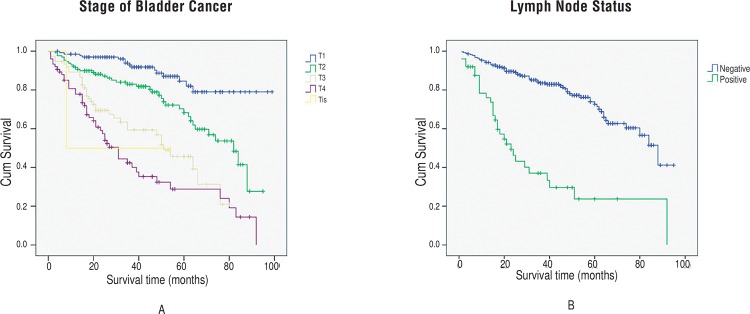
Estimated Kaplan-Meier overall survival stratified by stage of bladder cancer (a) and lymph node status (b) (all *P* <0.001).

Four hundred and thirty-six patients (57.1%) were successfully followed, and the median follow-up duration was 46.5 months (range 4–99 months). One hundred and sixty-seven patients died during follow-up, including 104 who died of bladder cancer and 63 patients died of other reasons, while no patients died of prostate cancer. The 5-year overall survival rate was 62.1%, with a median survival time of 80 months. The 5-year cancer–specific survival rate was 72%. According to a univariate analyses, incidental prostate cancer was not associated with cancer-specific survival (*P*=0.192) or overall survival (*P*=0.493). According to multivariate analyses, bladder cancer stage (*P*<0.001) and lymph node involvement (*P*<0.001) were independently associated with cancer-specific survival. Kaplan--Meier overall survival estimates, stratified by stage of bladder cancer and lymph node status are shown in Figure-1.

**Figure 2:**
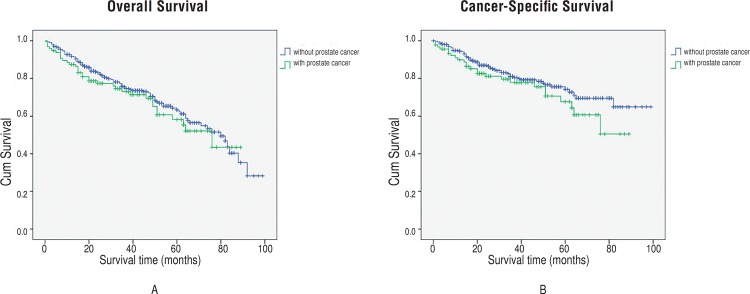
Estimated Kaplan-Meier overall survival curves (*P* =0.432) (a) and cancer-specific survival curves (*P* =0.190) (b) stratified by presence of prostate cancer.

**Figure 3:**
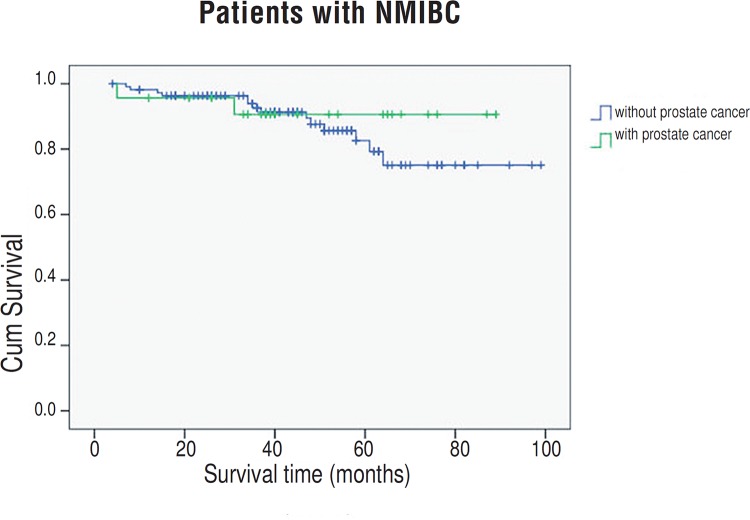
Estimated Kaplan-Meier overall survival curves for patients with non-muscle invasive bladder cancer stratified by presence of prostate cancer (*P* =0.510).

Ninety-six patients with prostate cancer were successfully followed, and none had experienced tumor recurrence (neither PSA recurrence, local recurrence, nor distant metastasis occurred). The 5-year overall survival rates for patients with or without prostate cancer were 58.2% and 63.3%, respectively, which showed no significant difference (*P*=0.432, Figure-2a). The 5-year cancer–specific survival rates for patients with or without prostate cancer were 67.6% and 74.2%, respectively, and no significant between-group difference was found (*P*=0.190, Figure-2b). When restricting analyses to non-muscle invasive bladder cancer only (NMIBC, including pTa, pT1, CIS), still no difference was found (*P*=0.510, Figure-3).

**Figure 4:**
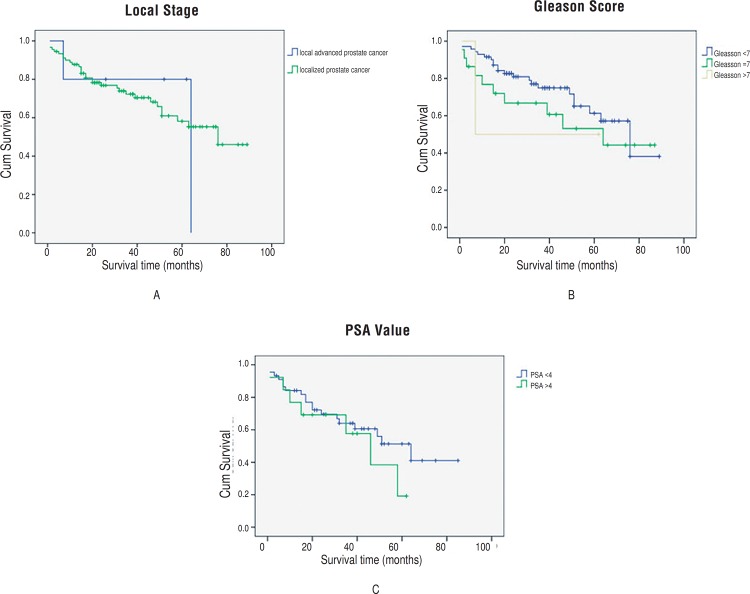
Estimated Kaplan-Meier overall survival curves for patients with prostate cancer stratified by stage of prostate cancer (*P*= 0.966) (a) Gleason score (*P*= 0.504) (b) and PSA value (*P*= 0.354) (c).

According to univariate analyses, the overall survival of patients with prostate cancer was not associated with prostate cancer stage, PSA value, or Gleason score (all *P* values>0.05). We grouped the patients based on stage of prostate cancer (localized prostate cancer [stage ≤pT2c], local advanced prostate cancer [stage ≥pT3a]), Gleason score (Gleason score<7, Gleason score=7, Gleason score>7) and PSA value (PSA< ng/mL and PSA≥4 ng/mL). Neither analysis showed any significant difference between groups in overall survival (all P>0.05, Figure-4).

## DISCUSSION

The incidence of prostate cancer found in cystoprostatectomy specimens in Western countries was reported to be 18%-50% (6-8), while in China, the reported incidence is much lower (3%-28%) (5, 9-11). Although prostate cancer is prevalent, it has comparatively low morbidity and mortality rates (12). A number of reports regarding incidental prostate cancer have been published, but no consensus about its impact on survival has been reached. Androulakakis et al. reported that incidental prostate cancer found during radical cystoprostatectomy did not affect the overall prognosis (13). Pritchett et al. had reported similar results (14). However, Konski reported that patients with prostate cancer had a better prognosis (15).

In this series, it appeared that the overall prognosis of patients with incidental prostate cancer might depend on the prognosis of bladder cancer. Whether the patient had concomitant prostate cancer did not decrease overall survival. The most important risk factor was bladder tumor stage and lymph node involvement. Furthermore, it is also notable that no patient died from prostate cancer, and none experienced PSA recurrence. In addition, for those patients with concomitant prostate cancer, those who had a higher pathologic stage, higher PSA level or higher Gleason score did not have a poorer prognosis. Concerning that conclusion mentioned above, the management of follow-up would not be necessary to be different from that of patients without incidental prostate cancer found in radical cystectomy, exclusion of monitoring serum PSA level.

Informed men requesting an early diagnosis in prostate cancer should be given a PSA test (16), and this important serum marker was a better predictor than either digital rectal examination (DRE) or transrectal ultrasound (TRUS) (17). PSA is an important marker during prostate cancer screening and follow-up. In China, the recommended cutoff value for prostate biopsy is 4ng/mL. In this series, the PSA value before operation was low; among those patients who had incidental prostate cancer, 80.3% had a PSA value below 4ng/mL. In our previous study, Yang et al. had reported that patients with incidental prostate cancer had a mean PSA of 3.28ng/mL before operation, while those without prostate cancer had a mean PSA of 2.18ng/mL (5). On the other hand, in the subgroup analysis of existing of incidental prostate cancer found in radical cystectomy, 61 (80.3%) patients had a PSA value below 4ng/mL versus the above ones, while there was no significant difference in overall survival (*P*=0.354). Thus, interestingly, it seems that PSA value before operation is not helpful for excluding incidental prostate cancers, and patients with higher PSA had similar survival results. According to the available data, PSA might not be a sole predictor of incidental prostate cancer, and there is still no consensus for the PSA cutoff value above which patients should have a prostate biopsy.

In this study, patients with incidental prostate cancer were older, which was in accordance with a European report by Pignot et al. (18). Recently, some investigators had reported a prostate-sparing approach to preserving sexual function for patients undergoing radical cystectomy (3, 19-22). This approach is only suitable in a highly selected population with bladder cancer without involvement of the prostatic urethra and without prostate cancer. This procedure is said to be oncologically safe with good functional results as long as it is performed in an experienced center (23). Our study might throw some light upon this issue. In addition to our conclusion, it was suggested that the selection of surgery method still needs some other consideration about prostate cancer screening comprehensively. Thus, a thorough examination of the prostate should be performed to preclude latent prostate cancer. However, from available data, there is still no effective predictor, especially PSA level studied in our research, to preclude prostate cancer before operation. Random prostate biopsy or transurethral resection of prostate may be indicated for those patients who wish to preserve their prostates, at least for patients with older age. Our conclusion might be an evidence of selecting prostate-sparing cystectomy or partial prostatectomy alternatively, which could protect sex function and maintain continence better. However, some related researches should be made to confirm it clearly in future studies.

This study has some limitations. The first limitation is the retrospective design of this study, which may lead to selection bias. Moreover, the volume of the prostate cancer was not exactly measured, which might have been informative to us. Furthermore, a pre-operative PSA value was not available for every patient. Finally, the patients who were lost to follow-up might have biased the survival analysis.

In conclusion, prostate cancer incidentally discovered at the time of radical cystectomy does not decrease overall survival. Patients with incidental prostate cancer were older than those without. PSA value before operation is not helpful for predicting incidental prostate cancers.
